# Excellent outcome after desensitization in high immunologic risk kidney transplantation

**DOI:** 10.1371/journal.pone.0222537

**Published:** 2019-09-24

**Authors:** Jeong-Hoon Lim, Jang-Hee Cho, Hee-Yeon Jung, Ji-Young Choi, Sun-Hee Park, Yong-Lim Kim, Hyung-Kee Kim, Seung Huh, Eun Sang Yoo, Dong-Il Won, Chan-Duck Kim

**Affiliations:** 1 Department of Internal Medicine, School of Medicine, Kyungpook National University, Kyungpook National University Hospital, Daegu, South Korea; 2 Department of Surgery, School of Medicine, Kyungpook National University, Kyungpook National University Hospital, Daegu, South Korea; 3 Department of Urology, School of Medicine, Kyungpook National University, Kyungpook National University Hospital, Daegu, South Korea; 4 Department of Clinical Pathology, School of Medicine, Kyungpook National University, Kyungpook National University Hospital, Daegu, South Korea; Postgraduate Medical Institute, INDIA

## Abstract

**Introduction:**

HLA-incompatible (HLAi) and ABO-incompatible (ABOi) kidney transplantation (KT) has been on the increase over the last decade. However, there are wide variations in outcomes from these procedures. In this study we evaluated the graft and patient outcomes in incompatible KT and non-sensitized KT.

**Methods:**

Patients who underwent KT between January 2012 and April 2018 were enrolled and reviewed. We divided kidney transplant recipients (KTRs) into five groups as follows: HLAi (*n* = 50); ABOi (*n* = 65); HLAi+ABOi (*n* = 5); control (*n* = 428); and living-donor control (LD control, *n* = 218). We compared the risk of rejection, graft function, graft survival, and patient survival between incompatible KTRs and control/LD control KTRs.

**Results:**

Although the incidence of active antibody-mediated rejection in HLAi group tends to be higher than in control and LD control groups (6.0% vs. 2.8%, *P* = 0.20; 6.0% vs. 3.7%, *P* = 0.44, respectively), the rejection-free survival, graft survival, and patient survival were not significantly different from those of the control and LD control groups in all three incompatible KT groups (all *P*>0.05). Graft function during the study period was also not different between incompatible KTRs and control/LD control groups (both *P*>0.05). Using Cox regression analysis, neither HLAi nor ABOi were risk factors for graft failure. Some infectious diseases such as urinary tract infection and cytomegalovirus infection were more common in the HLAi group than in the control/LD control group (both *P*<0.05), but only one infection-related death occurred in HLAi KTRs. Infection risks were similar in the ABOi and HLAi+ABOi groups compared to controls.

**Conclusion:**

Our results showed favorable outcomes for incompatible KT after desensitization. Although desensitization therapy for incompatible KT has improved access to transplantation for KT candidates with high immunological risk, more clinical data are clearly needed.

## Introduction

Kidney transplantation (KT) is the treatment of choice for the most end-stage renal disease (ESRD) patients [1, 2]. However, donated kidneys are still scarce, and many ESRD patients around the world are waiting for transplants [3]. To address this issue, HLA-incompatible (HLAi) and ABO-incompatible (ABOi) KT were introduced, and the use of these techniques has been on the increase over the last decade.

Both HLAi and ABOi KT recipients (KTRs) have incompatible antibodies before KT: anti-HLA donor-specific antibodies (DSAs) and anti-A/B antibodies, respectively. The incompatible antibodies are removed in a multi-step process before transplant. These desensitization methods are similar for both types of incompatible KT, and include the use of plasmapheresis, rituximab, and intravenous immunoglobulin (IVIG). In spite of the desensitization and removal of antibodies before transplant, several studies have reported that rejection and graft failure risks are higher in HLAi KTRs than in ABOi or in non-sensitized KTRs [4–8]. These studies suggested the importance of DSA levels rather than anti-A/B antibody levels in rejection and graft failure [7, 9]. In particular, DSAs are associated with a higher incidence of active antibody-mediated rejection (ABMR) [4, 10].

The extent of the risk of rejection and graft failure in incompatible KTs is still controversial. There are no consistent reports on rejection rates and graft failure rates in sensitized KT. Study results have shown wide variation by transplant center and country [6–9] making it difficult to precisely judge risks in transplants. To help patients and clinicians better understand and predict outcomes for incompatible KT, we investigated and compared the clinical outcomes of HLAi, ABOi, combined HLAi and ABOi (HLAi+ABOi), and compatible KT.

## Materials and methods

### Study populations

**Fig 1:**
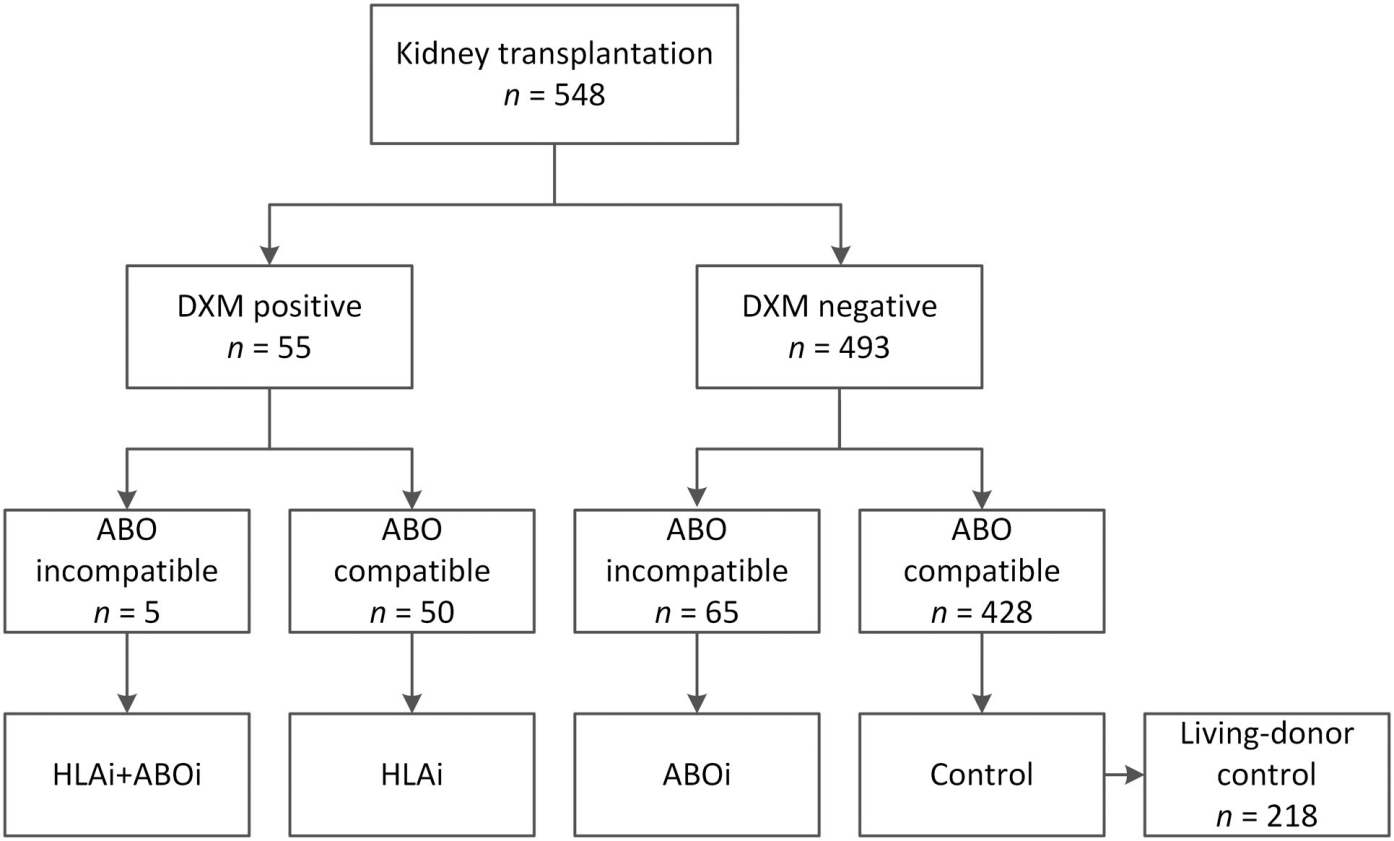
Baseline patient distribution according to ABO and immunological matches. Abbreviations: ABOi, ABO incompatible; DXM, direct cross-match; HLAi, HLA incompatible; LD, living-donor.

Patients who underwent KT in Kyungpook National University Hospital between January 2012 and April 2018 were analyzed retrospectively. We divided KTRs into five groups, according to cross-matching results and ABO incompatibility with donors (Fig 1). Patients who had positive complement-dependent cytotoxicity cross-matches (CDC-XM) or flow cytometric cross-matches (FCXM; either T or B cell) before KT were defined as HLAi KTRs. ABOi KT was defined as KT from ABO-incompatible donors. The patients who met both HLAi and ABOi KT criteria were classified into the HLAi+ABOi group. Among the control group, the patients who underwent living-donor kidney transplant were further classified as living-donor control group (LD control). The study protocol was reviewed and was approved by the Institutional Review Board of Kyungpook National University Hospital (2018-12-012). The informed consent was waived since the study was conducted by retrospective review of medical records. All patient information were anonymized and they were de-identified before analyses.

### Immunologic identification before kidney transplantation

All KTRs were immunologically evaluated before transplant. The details of the immunologic work-up are as follows: (1) T and B cell cross-matching for both CDC-XM and FCXM; (2) panel-reactive antibody (PRA) screening using the Luminex method; (3) if either (1) or (2) was positive, PRA identification (ID) or Luminex single-antigen assay (LSA) was carried out to identify DSA; (4) in the case of ABOi KT, Anti-A and anti-B antibody titers were measured using the column agglutination technique (CAT) [11]; (5) HLA-A, B, DR, and DQ typing were performed at the DNA level in both recipients and donors. We used the Luminex method for PRA, and the positive threshold set for mean fluorescence intensity (MFI) was above 1,000.

### Desensitization protocols

Our center’s desensitization protocol included the use of rituximab, plasmapheresis, IVIG, and optionally induction anti-thymocyte globulin (ATG). In HLAi and ABOi LDKT with a general risk, a 200mg or 500mg fixed dose of rituximab was administered three weeks before transplant. Plasmapheresis was initiated 10 to 14 days before KT, and performed every other day. After each plasmapheresis, low dose IVIG (0.1g/kg) was injected. In the case of HLAi KT from deceased donor, individualized desensitization treatment including plasmapheresis, high-dose IVIG (1.0g/kg), rituximab, and ATG induction, were applied considering each patient’s general health condition, infection risk, morbidity, post-KT complication such as bleeding, socioeconomic status, and degree of sensitization to donor. For induction therapy, 20mg basiliximab was administered intravenously on days zero and four after KT. For patients with immunologic risk, we used 1.0–1.5g/kg ATG for three days (days zero to two) instead of basiliximab. For HLAi KTRs, our target was to achieve negative cross-matching and/or negative DSA MFI level (<3,000). After rituximab injection, only DSA MFI level was applied to the target because of the false positive result of B cell FCXM [12]. For ABOi KTRs, our target was to achieve an anti-ABO antibody titer before transplantation of less than 1:16.

### Immunosuppressive regimen

We applied standard triple immunosuppressive therapy to KTRs. Tacrolimus, mycophenolate mofetil, and methylprednisolone were started 10–14 days before KT. The target tacrolimus trough level was 8ng/mL to 12ng/mL for the first month after KT, which was then tapered to between 5ng/mL and 8ng/mL. Mycophenolate mofetil was prescribed daily at a fixed dose of 1.0–2.0g. Intravenous methylprednisolone (500mg) was administered at the time of surgery and was tapered to five milligrams per day within six months after KT.

### Post-transplant follow-up schedule

All patients without post-transplant complication were discharged at 2 weeks after KT and regularly visited the outpatient nephrology clinic. Detailed visit schedule is as follows: (1) weekly visit for the first 3 months after KT; (2) biweekly visit for the next 3 months (3–6 months after KT); (3) monthly visit after 6 months after KT.

### Clinical outcomes and parameters

We investigated clinical outcome factors including estimated glomerular filtration rate (eGFR), biopsy-proven acute rejection (BPAR) rate, graft survival, and patient survival. In addition, cause of death, incidence of infectious disease, and risk factors for graft failure were evaluated. eGFR was measured at two weeks, and one, two, three, four, and five years after transplant, as calculated using the Chronic Kidney Disease Epidemiology Collaboration equation [13]. BPAR was diagnosed based on the Banff classification [14], and was subdivided according to the time of diagnosis (early: one year or less after transplant, and late: more than one year after transplant), and type of rejection (acute T-cell mediated rejection [TCMR] and active ABMR). Graft survival was defined as the time from KT to re-start of the renal replacement therapy. In the case of the patient death with a functioning graft, the patient’s graft survival was censored at the time of death. Patient survival was defined as the time from KT to death from any cause. The clinical outcome results of each incompatible KT were compared to those from non-sensitized KT.

We defined delayed graft function in individuals who needed dialysis during the first week after KT because of poor graft function [15]. Cytomegalovirus (CMV) infection was defined in KTRs who had CMV antigenemia or who were positive for CMV polymerase chain reaction, and were also treated for CMV. BK virus nephropathy was defined as KTRs who had more than 10,000 copies/mL of BK virus DNA in serum and were treated for BK virus, or in whom BK virus nephropathy was diagnosed by graft biopsy [16].

### Statistical analysis

Continuous variables were summarized as mean ± SD or median (range), and categorical variables were presented as number and percentage (%). Student’s *t*-tests were used to assess differences between continuous variables, and Pearson chi-square tests or Fisher’s exact tests were used to investigate differences between categorical variables. Rejection-free survival, graft survival, and patient survival were analyzed using Kaplan-Meier analysis, and the differences with those of control patients were analyzed using log-rank tests. To identify the risk factors for graft failure for all KTRs, univariate and multivariate Cox proportional hazard regression analysis was applied. Variables that showed significant differences using univariate analysis and had possible confounding factors (HLAi and ABOi) were subjected to a multivariate model. Statistical analysis was performed using SPSS version 20.0 (SPSS Inc., Chicago, IL, USA) and R (R Foundation for Statistical Computing, Vienna, Austria; www.r-project.org). *P* values less than 0.05 were considered to be statistically significant.

## Results

### Baseline characteristics

During the study period, there were 548 KTRs. Of these, 50 patients had HLAi KT, 65 had ABOi KT, 5 had HLAi+ABOi KT, and the remaining 428 formed the control group. Among the control group, 218 KTRs underwent LDKT and they were further classified as LD control group. We analyzed the baseline characteristics of each patient group (Table 1). The mean age was similar between HLAi, ABOi, HLAi+ABOi, and control groups (*P*>0.05), but LD control group was younger than HLAi group (*P* = 0.014). The proportion of female patients was higher in the HLAi KT group compared with control and LD control KTRs (86.0% vs. 34.6%, *P*<0.001; 86.0% vs. 34.1%, *P*<0.001, respectively). The mean follow-up period after transplant were 35.6 months in the control group and 35.1 months in the LD control group, and were significantly longer in the HLAi KT group than in both control and LD control group (43.7 months; *P* = 0.036 and *P* = 0.030, respectively). Glomerulonephritis was the most common primary renal disease in all groups. The re-transplant ratio was 12.0% in the HLAi group, higher than in both control and LD control group (*P* = 0.012 and *P* = 0.004). All incompatible groups—HLAi, ABOi, and HLAi+ABOi—had higher proportions of living donor KT than the control group.

**Table 1:** Baseline characteristics.

	HLAi (*n* = 50)	ABOi (*n* = 65)	HLAi+ABOi (*n* = 5)	Control (*n* = 428)	LD control (*n* = 218)
Age (y)	49.0 ± 8.3^†^	48.1 ± 12.5	46.4 ± 11.5	47.7 ± 12.3	45.4 ± 12.8
Gender, female (%)	43 (86.0)*^,^ ^†^	15 (23.1)	3 (60.0)	148 (34.6)	74 (34.1)
Body mass index	21.2 ± 2.8*^,^ ^†^	22.4 ± 3.4	20.9 ± 1.1	22.4 ± 3.5	22.6 ± 3.8
Follow up after KT (mo)	43.7 ± 25.1*^,^ ^†^	35.3 ± 21.4	32.1 ± 25.0	35.6 ± 20.4	35.1 ± 20.3
Dialysis vintage before KT (mo)	53.1 ± 79.2^†^	8.3 ± 18.3*	3.9 ± 6.3*	51.6 ± 86.9	9.0 ± 26.2
Primary renal disease, n (%)					
Glomerulonephritis	33 (66.0)	33 (50.8)	5 (100.0)	231 (54.0)	114 (52.3)
Diabetes mellitus	11 (22.0)	23 (35.4)	0 (0.0)	131 (30.6)	68 (31.2)
Hypertension	2 (4.0)	5 (7.7)	0 (0.0)	30 (7.0)	15 (6.9)
Polycystic kidney disease	1 (2.0)	1 (1.5)	0 (0.0)	15 (3.5)	10 (4.6)
Others	3 (6.0)	3 (4.6)	0 (0.0)	21 (4.9)	11 (5.0)
Comorbid conditions, n (%)					
Hypertension	13 (26.5)	8 (12.3)	0 (0.0)	83 (19.4)	42 (19.3)
Diabetes mellitus	37 (74.0)	37 (56.9)	4 (80.0)	286 (66.8)	145 (66.5)
Re-transplant, n (%)	6 (12.0)*^,^ ^†^	5 (7.7)^†^	1 (20.0)	14 (3.3)	4 (1.8)
Number of HLA mismatch	3.4 ± 1.3*	3.4 ± 1.8*	3.6 ± 1.1	2.9 ± 1.7	3.0 ± 1.6
Number of HLA-DR mismatch	1.2 ± 0.6*^,^ ^†^	1.2 ± 0.7*	1.2 ± 0.5	1.0 ± 0.7	1.0 ± 0.7
Delayed graft function, n (%)	8 (16.0)^†^	0 (0.0)*	0 (0.0)	44 (10.3)	2 (0.9)
Donor age (y)	43.0 ± 14.5^†^	46.6 ± 11.7	42.2 ± 10.0	47.4 ± 14.5	48.3 ± 12.1
Donor gender (% male)	32 (64.0)^†^	25 (38.5)*	3 (60.0)	231 (54.0)	95 (43.6)
Donor body mass index	23.3 ± 3.6	23.4 ± 3.2^†^	25.1 ± 6.1	23.6 ± 3.4	24.3 ± 3.1
Transplantation type, n (%)	*^,^ ^†^	*^,^ ^†^	*		
Living-related	20 (40.0)	29 (44.6)	2 (40.0)	141 (32.9)	141 (64.7)
Living-unrelated	15 (30.0)	36 (55.4)	3 (60.0)	77 (18.0)	77 (35.3)
Deceased	15 (30.0)	0 (0.0)	0 (0.0)	210 (49.1)	0 (0.0)

*P<0.05 vs. control.

^†^*P*<0.05 vs. LD control.

Continuous variables are shown as means ± standard deviation.

Abbreviations: HLAi, HLA-incompatible; ABOi, ABO-incompatible; LD, living-donor; KT, kidney transplantation.

### Immunological characteristics

Baseline immunological information is shown in Table 2. The proportion of patients with HLA class I and II DSAs was similar between the HLAi and HLAi+ABOi groups (class I: 42.0% vs. 40.0%; class II: 56.0% vs. 60.0%; both *P*>0.05). After desensitization, all DSA MFIs were less than 3,000. All HLAi and HLAi+ABOi patients showed FCXM positive at baseline, and CDC-XM positive rates were 24.0% and 20.0%, respectively. In the ABOi and HLAi+ABOi patients, the median baseline anti-ABO IgG titers were 1:16 and 1:32, respectively, and the ranges of both were 2 to 1024 at baseline. After desensitization, median titers decreased to 4 and 8, respectively, and the ranges of both were 1 to 32. The ratio of patients who underwent plasmapheresis was lower in the HLAi group than in the ABOi group (*P*<0.05). HLAi+ABOi patients underwent more sessions of plasmapheresis than HLAi or ABOi patients (both *P*<0.05). More ABOi patients used a lower dose of rituximab (200mg) compared with HLAi and HLAi+ABOi patients (both *P*<0.05). All ABOi patients used basiliximab as induction therapy, but 54.0% of HLAi group and 80.0% of the HLAi+ABOi group used ATG as induction therapy (both *P*<0.05, compared to the ABOi group). Among the control patients, 405 (94.6%) patients used basiliximab and 23 (5.4%) used ATG as induction therapy. In the LD control group, 214 (98.2%) patients used basiliximab and 4 (1.8%) used ATG as induction therapy.

**Table 2:** Immunologic characteristics.

	HLAi (*n* = 50)	ABOi (*n* = 65)	HLAi+ABOi (*n* = 5)
HLA-DSA			
Class I DSA, n (%)	21 (42.0)		2 (40.0)
Baseline class I DSA MFI, median (range)	2118 (1208−6867)		4715 (1207−8222)
Baseline class I DSA MFI, median (IQR)	2118 (1909, 3550)		
Pre-transplant class I DSA MFI, median (range)	410 (0−2997)		1299 (0−2597)
Class II DSA, n (%)	28 (56.0)		3 (60.0)
Baseline class II DSA MFI, median (range)	3031 (1042−19899)		2113 (1485−5120)
Baseline class II DSA MFI, median (IQR)	3031 (2262, 9221)		
Pre-transplant class II DSA MFI, median (range)	859 (95−2999)		222 (0−2597)
Direct crossmatch			
FCXM, n (%)	50 (100.0)		5 (100.0)
FCXM T cell, n (%)	22 (44.0)		4 (80.0)
FCXM T cell MFI ratio, median (range)	3.30 (1.80, 10.20)		2.75 (2.30, 4.70)
FCXM B cell, n (%)	47 (94.0)		5 (100.0)
FCXM B cell MFI ratio, median (range)	4.55 (2.00, 78.20)		13.20 (4.00, 15.50)
CDC-XM, n (%)	12 (24.0)		1 (20.0)
CDC-XM T cell, n (%)	4 (8.0)		0 (0.0)
CDC-XM B cell, n (%)	11 (22.0)		1 (20.0)
ABO isoagglutinin titer, median (range)			
Baseline		16 (2, 1024)	32 (2, 1024)
Pre-transplant		4 (1, 32)	8 (1, 32)
Desensitization			
Pre-transplant plasmapheresis, n (%)	35 (70.0)^†^	64 (98.5)*	5 (100.0)
Post-transplant plasmapheresis, n (%)	4 (8.0)^†^	0 (0.0)*	0 (0.0)
Number of plasmapheresis	3.66 ± 2.63^‡^	3.49 ± 2.13^‡^	6.40 ± 2.79*^,^ ^†^
Rituximab, n (%)	45 (90.0)	65 (100.0)	5 (100.0)
Dose of rituximab, 200 mg	8 (16.0)^†^	51 (78.5)*^,^ ^‡^	0 (0.0)^†^
Dose of rituximab, 500 mg	37 (74.0)^†^	14 (21.5)*^,^ ^‡^	5 (100.0)^†^
IVIG, n (%)	45 (90.0)	64 (98.5)	5 (100.0)
Induction therapy			
Basiliximab, n (%)	23 (46.0)^†^	65 (100.0)*^,^ ^‡^	1 (20.0)^†^
Anti-thymocyte globulin, n (%)	27 (54.0)^†^	0 (0.0)*^,^ ^‡^	4 (80.0)^†^

*P<0.05 vs. HLAi;

^†^*P*<0.05 vs. ABOi;

^‡^*P*<0.05 vs. HLAi+ABOi.

Continuous variables are shown as means ± standard deviation or medians (range).

Abbreviations: HLAi, HLA-incompatible; ABOi, ABO-incompatible; DSA, donor-specific antibody; MFI, mean fluorescence intensity; IQR, inter-quartile range; FCXM, flow cytometric cross-match; CDC-XM, complement-dependent cytotoxicity cross-match; IVIG, intravenous immunoglobulin.

### Comparison of rejection rates

The incidence of BPAR was 6.0% (3 of 50) in the HLAi group, 6.2% (4 of 65) in the ABOi group, 0% (0 of 5) in the HLAi+ABOi group, 7.5% (32 of 428) in the control group, and 8.3% (18 of 218) in the LD control group. The proportion was not different between groups (all *P*>0.05) (Table 3). There were no rejection episodes in the HLAi+ABOi group during follow-up. The rate of active ABMR was the highest in the HLAi group (6.0%, 3 of 50), but there was no statistical difference compared to the control and LD control group (*P* = 0.200 and *P* = 0.435, respectively). Two active ABMR episodes, defined as early active ABMR, occurred within one year after transplant in the HLAi group (4.0%, 2 of 50), four occurred in the control group (0.9%, 4 of 428), and three occurred in the LD control group (1.4%, 3 of 218). All of the KTRs with early active ABMR showed *de novo* DSA at the time of diagnosis. Among BPAR, there was one patient with mixed acute TCMR and active ABMR in the HLAi group, two in the ABOi group, five in the control group, and two in the LD control group. The proportion of acute TCMR was one of three in the HLAi group, four of four in the ABOi group, 25 of 32 in the control group, and 12 of 18 in the LD control group.

**Table 3:** Comparison of rejection.

*n* (%)	HLAi (*n* = 50)	ABOi (*n* = 65)	HLAi+ABOi (*n* = 5)	Control (*n* = 428)	LD control (*n* = 218)
Active ABMR	3 (6.0)	2 (3.1)	0 (0.0)	12 (2.8)	8 (3.7)
Early	2 (4.0)	0 (0.0)	0 (0.0)	4 (0.9)	3 (1.4)
Late	1 (2.0)	2 (3.1)	0 (0.0)	8 (1.9)	5 (2.3)
Acute TCMR	1 (2.0)	4 (6.2)	0 (0.0)	25 (5.8)	12 (5.5)
Early	0 (0.0)	2 (3.1)	0 (0.0)	14 (3.3)	6 (2.8)
Late	1 (2.0)	2 (3.1)	0 (0.0)	11 (2.6)	6 (2.8)
BPAR^†^	3 (6.0)	4 (6.2)	0 (0.0)	32 (7.5)	18 (8.3)
Early	2 (4.0)	2 (3.1)	0 (0.0)	16 (3.7)	8 (3.7)
Late	1 (2.0)	2 (3.1)	0 (0.0)	16 (3.7)	10 (4.6)

^†^There was one mixed acute TCMR and active ABMR in HLAi group, two in ABOi group, five in control group, and two in LD control group.

All *P* values exceeded 0.05 when compared to control and LD control group. Early indicates within 1 year and late indicates after 1 year after kidney transplantation.

Abbreviations: HLAi, HLA-incompatible; ABOi, ABO-incompatible; LD, living-donor; ABMR, antibody-mediated rejection; TCMR, T-cell mediated rejection; BPAR, biopsy-proven acute rejection.

**Fig 2:**
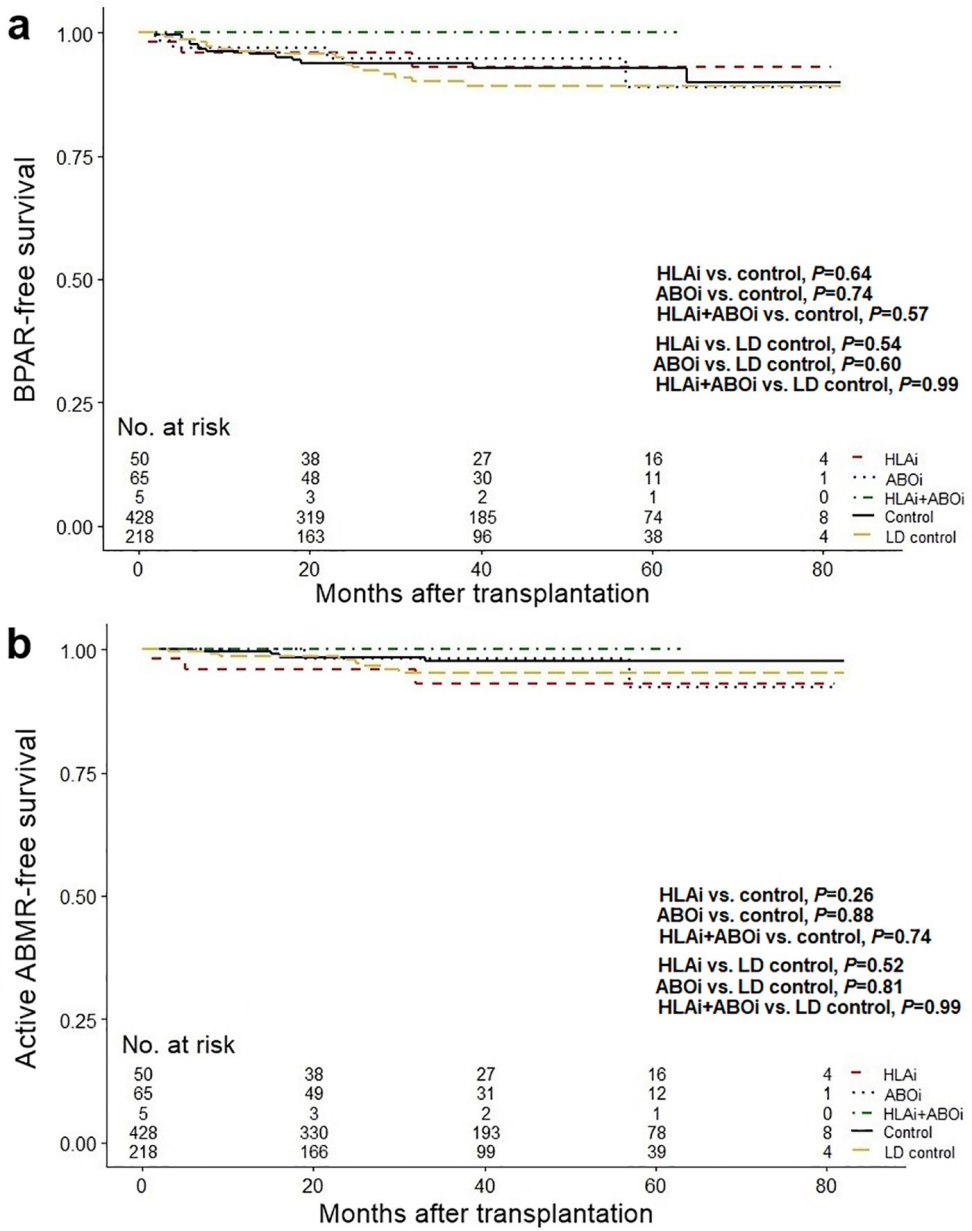
Comparison of rejection-free survival. (a) Biopsy-proven acute rejection free survival in HLAi, ABOi, HLAi+ABOi, control, and LD control groups. (b) Active antibody-mediated rejection free survival in HLAi, ABOi, HLAi+ABOi, control, and LD control groups. Abbreviations: ABOi, ABO-incompatible; ABMR, antibody-mediated rejection; BPAR, biopsy-proven acute rejection; HLAi, HLA-incompatible; LD, living-donor.

Fig 2 shows BPAR- and active ABMR-free survival for each group. The HLAi, ABOi, and HLAi+ABOi groups showed no decrease in BPAR- and active ABMR-free survival compared to the control group and LD control group (all *P*>0.05).

### Comparison of graft survival and allograft function

**Fig 3:**
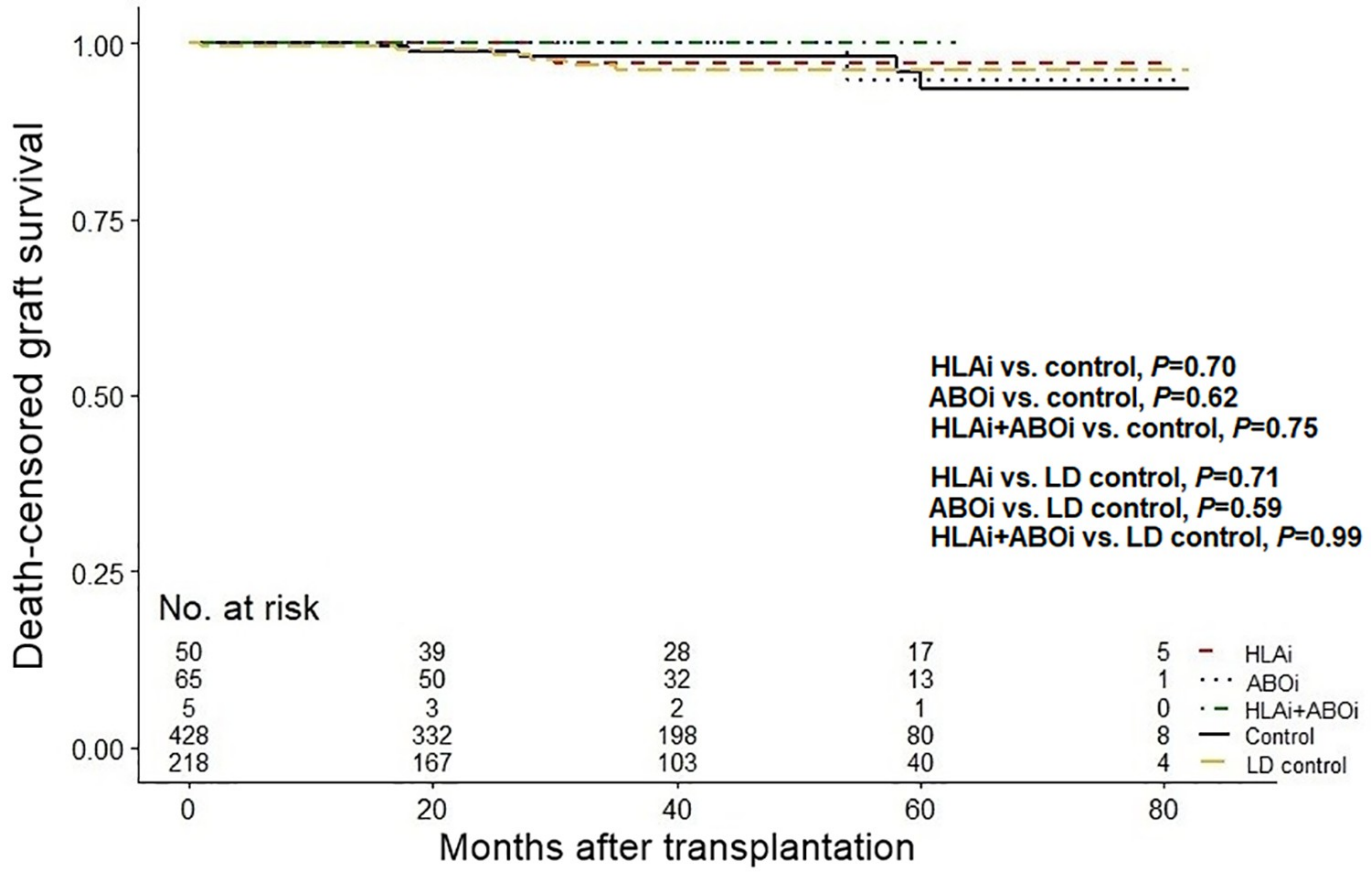
Comparison of death-censored graft survival. Abbreviations: ABOi, ABO-incompatible; HLAi, HLA-incompatible; LD, living-donor.

The number of patients who exhibited graft failure was one in the HLAi group (2.0%), one in the ABOi group (1.5%), none in the HLAi+ABOi group (0%), 11 in the control group (2.6%), and six in the LD control group (2.8%). Death-censored graft survival is depicted in Fig 3. There was no significant differences in graft survival in any of the three incompatible groups compared to the control group and LD control group (all *P*>0.05).

**Fig 4:**
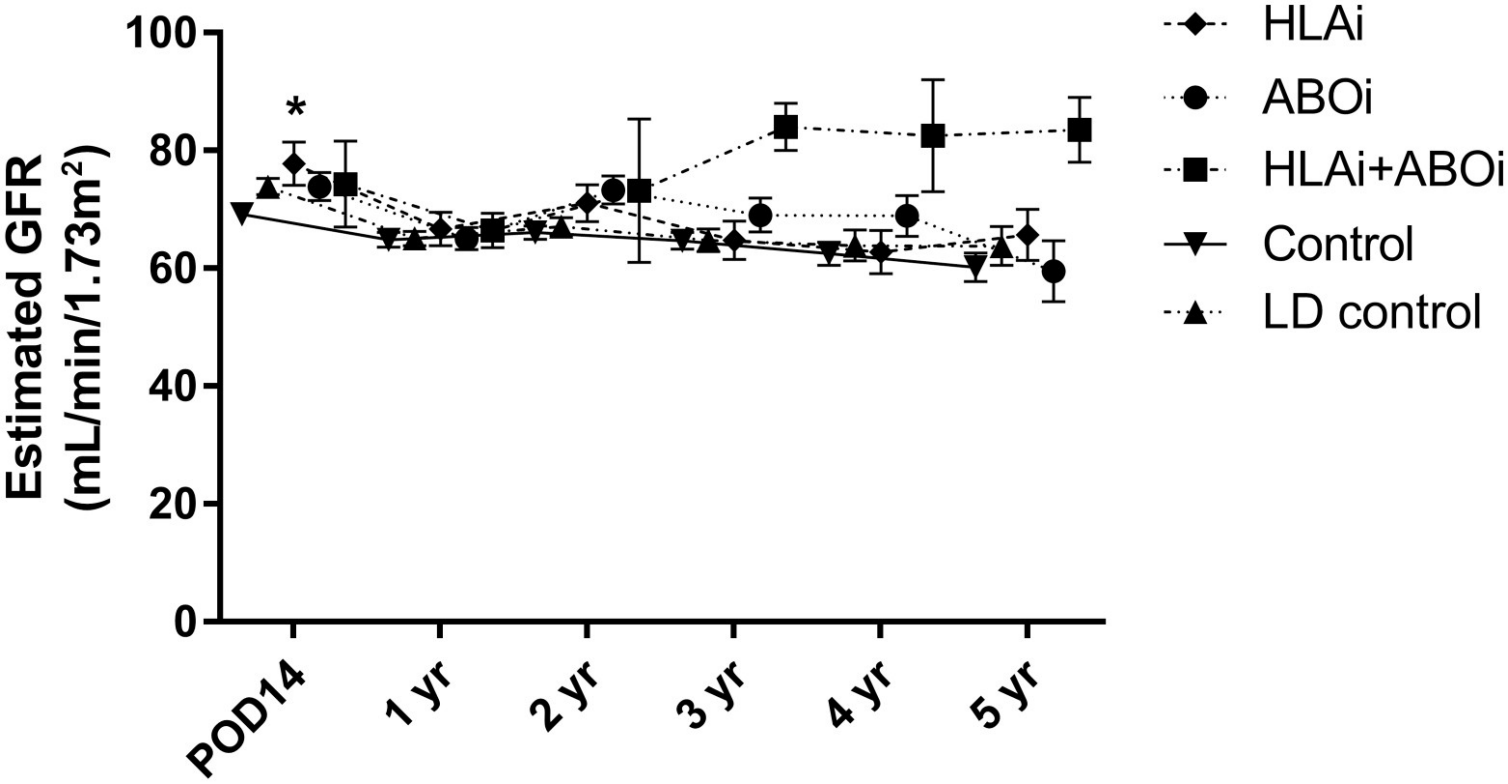
Comparison of serial changes of graft function. *P<0.05 versus control. Abbreviations: ABOi, ABO-incompatible; GFR, glomerular filtration rate; HLAi, HLA-incompatible; LD, living-donor.

Serial changes in the eGFR in each patient group are shown in Fig 4. At 14 days after transplant, eGFR was significantly higher in the HLAi group than in the control group (77.8mL/min/1.73m^2^ vs. 69.2mL/min/1.73m^2^, *P* = 0.015). There were no other differences in eGFR in the HLAi, ABOi, or HLAi+ABOi groups compared to the control group and LD control group during five years of follow-up (all *P*>0.05).

A Cox proportional hazards model analysis was used to identify risk factors for graft failure (Table 4). In a univariate analysis, obesity, high HLA mismatch numbers, increased age of donor, occurrence of any kind of rejection, and reduced eGFR at one year after KT were related to decreased graft survival (all *P*<0.05). Amongst these factors, a donor age of 60 years or more, and the occurrence of active ABMR or acute TCMR were identified as independent risk factors for graft failure in a multivariate analysis (hazard ratio [HR], 8.93 [95% confidence interval (CI), 1.73–46.00], *P* = 0.009; HR, 16.24 [95% CI, 2.74–96.24], *P* = 0.002; HR, 30.84 [95% CI, 6.80–139.97], *P*<0.001, respectively). HLAi and ABOi were not risk factors for graft failure (all *P*>0.05).

**Table 4:** Cox regression analysis for graft failure.

Variables	Univariate		Multivariate	
	HR (CI 95%)	*P*	HR (CI 95%)	*P*
HLA incompatible	0.63 (0.08–4.90)	0.662	0.44 (0.02–8.21)	0.581
ABO incompatible	0.60 (0.08–4.59)	0.620	0.09 (0.01–1.50)	0.093
Age ≥60 y	0.54 (0.07–4.14)	0.551		
Gender, male	1.31 (0.40–4.24)	0.658		
Preemptive transplant	1.48 (0.45–4.80)	0.519		
Hypertension	1.52 (0.34–6.86)	0.587		
Diabetes mellitus	0.99 (0.31–3.23)	0.993		
Primary GN	1.17 (0.38–3.58)	0.782		
BMI ≥25 kg/m^2^	4.11 (1.38–12.25)	0.011	1.48 (0.38–5.82)	0.572
HLA mismatches ≥5	3.13 (1.05–9.32)	0.040	0.44 (0.11–1.84)	0.262
HLA-DR mismatches >1	2.67 (0.90–7.94)	0.079		
Donor age ≥60 y	4.38 (1.47–13.04)	0.008	8.93 (1.73–46.00)	0.009
Donor gender, male	0.58 (0.19–1.77)	0.337		
Deceased donor KT	1.16 (0.39–3.46)	0.787		
BPAR	142.87 (18.56–1099.83)	<0.001		
Active ABMR	26.51 (8.90–78.94)	<0.001	16.24 (2.74–96.24)	0.002
Acute TCMR	50.14 (13.77–182.58)	<0.001	30.84 (6.80–139.97)	<0.001
1 year eGFR <60 mL/min/1.73m^2^	8.61 (1.91–38.92)	0.005	2.14 (0.30–15.57)	0.451
Re-transplant	1.95 (0.25–15.01)	0.524		

Abbreviations: HR, hazard ratio; CI, confidence interval; GN, glomerulonephritis; BMI, body mass index; KT, kidney transplantation; BPAR, biopsy-proven acute rejection; ABMR, antibody-mediated rejection; TCMR, T-cell mediated rejection; eGFR, estimated glomerular filtration rate.

### Comparison of infection complications and patient survival

Incidences of infectious disease are shown in Table 5. The incidence of urinary tract infection was higher in the HLAi group compared to the control group and LD control group (18.0% vs. 9.1%, *P* = 0.048; 18.0% vs. 7.8%, *P* = 0.036, respectively), and CMV infections were also more common in the HLAi group than in the control group and LD control group (10.0% vs. 1.9%, *P* = 0.007; 10.0% vs. 1.4%, *P* = 0.007, respectively). No infections occurred in the HLAi+ABOi patients. There was no difference in the incidence of other infections, including BK virus nephropathy, in any of the incompatible groups compared to the control group (all *P*>0.05).

**Table 5:** Causes of infection and death.

*n* (%)	HLAi (*n* = 50)	ABOi (*n* = 65)	HLAi+ABOi (*n* = 5)	Control (*n* = 428)	LD control (*n* = 218)
Bacterial infections					
Urinary tract infection	9 (18.0)*^,^ ^†^	10 (15.4)	0 (0.0)	39 (9.1)	17 (7.8)
Pneumonia	1 (2.0)	3 (4.6)	0 (0.0)	10 (2.3)	5 (2.3)
Cellulitis	1 (2.0)	1 (1.5)	0 (0.0)	5 (1.2)	1 (0.5)
Infectious enteritis	1 (2.0)	1 (1.5)	0 (0.0)	5 (1.2)	3 (1.4)
Others^a^	1 (2.0)	1 (1.5)	0 (0.0)	4 (0.9)	2 (0.9)
Viral infections					
CMV infection	5 (10.0)*^,^ ^†^	1 (1.5)	0 (0.0)	8 (1.9)	3 (1.4)
BK virus nephropathy	8 (16.0)	7 (10.8)	0 (0.0)	49 (11.4)	24 (11.0)
VZV infection	1 (2.0)	2 (3.1)	0 (0.0)	2 (0.5)	1 (0.5)
Fungal infections					
Pneumocystis infection	2 (4.0)	0 (0.0)	0 (0.0)	3 (0.7)	2 (0.9)
Candida infection	0 (0.0)	1 (1.5)	0 (0.0)	0 (0.0)	0 (0.0)
Death					
Infection	1 (2.0)	1 (1.5)	0 (0.0)	2 (0.5)	2 (0.9)
Cancer	1 (2.0)	0 (0.0)	0 (0.0)	1 (0.2)	0 (0.0)
Cardiovascular disease	0 (0.0)	0 (0.0)	0 (0.0)	1 (0.2)	0 (0.0)

^a^Others included tuberculosis, osteomyelitis, and pseudomembranous colitis.

*P<0.05 vs. control.

^†^*P*<0.05 vs. LD control.

Abbreviations: HLAi, HLA-incompatible; ABOi, ABO-incompatible; LD, living-donor; CMV, cytomegalovirus; VZV, varicella zoster virus.

**Fig 5:**
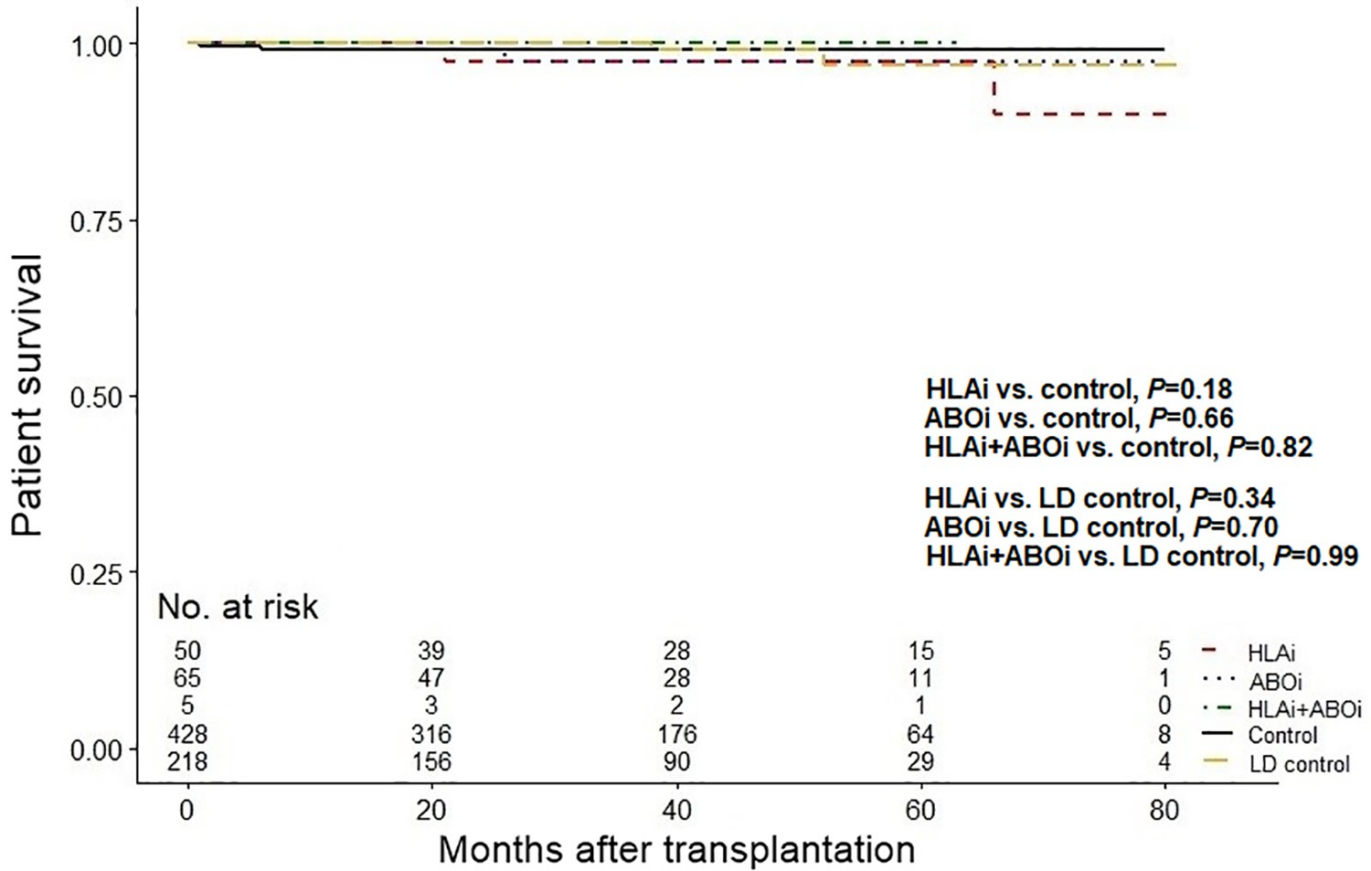
Comparison of patient survival. Abbreviations: ABOi, ABO-incompatible; HLAi, HLA-incompatible; LD, living-donor.

During the observation period there were two deaths in the HLAi group, one death in the ABOi group, no deaths in HLAi+ABOi group, four deaths in the control group, and two deaths in the LD control group (Table 5). Causes of death included infections, cancer, and cardiovascular disease. The incidence and cause of death were similar for all groups (all *P*>0.05). There was no significant difference in patient survival in the three incompatible groups compared with the control group and LD control group (all *P*>0.05, Fig 5).

## Discussion

Our study shows that the prognoses of any of the kinds of incompatible KT are not worse than those of compatible KT. We compared prognostic factors such as graft function, graft survival, patient survival, and rejection rates between incompatible and compatible KTRs, and none of the differences were significant. These results suggest that HLAi, ABOi, and HLAi+ABOi KTs can be a good choice for patients awaiting transplants with ESRD.

Similar desensitization protocols including rituximab, plasmapheresis, and IVIG have applied to incompatible KTs in many transplant centers [9, 17–21]. Nonetheless, the prognosis varies, a phenomenon which might be related to different pre-transplant target antibody levels, strengths of immunosuppression, physician proficiency, and ethnicity. The ideal safe ranges of pre-transplant DSA and anti-A/B antibody levels are still not clear. We used a pre-transplant DSA MFI target of less than 3,000 in HLAi KT and anti-A/B antibody titer of less than 16 in ABOi KT. In spite of following our desensitization protocol, one patient undergoing ABOi KT and one patient with HLAi+ABOi KT exceeded the allowed range, with both pre-transplant anti-A/B antibody titers of 32, but all HLAi KTRs met the target DSA level. We prescribed individualized immunosuppressive drugs taking into account the patients’ immunological risk, and we closely monitored trough levels and patient compliance. Since 2012, 22% of KTs performed in our center have been incompatible KTs, so we have accumulated significant experience. In addition to that, Asian KTRs showed better graft outcome in previous studies [22–24], so there may also have been an impact of ethnic and genetic differences in our results. Korean ethnic and national characteristics, including familism to provide emotional and financial support to KT recipients, healthful food culture with low obesity rates, and low immunosuppressant cost covered by national health insurance, may have influenced the excellent outcome.

As with other studies, the proportion of female patients was higher in the HLAi group than in the control group [7–9]. This difference might be due to sensitization from pregnancy [25, 26]. The lower BMI observed in the HLAi KTRs is probably also related to the higher proportion of women. Dialysis vintage was significantly shorter in the ABOi and HLAi+ABOi groups than in the control group, indicating that different blood types are no longer a serious hurdle to KT. Re-transplantation patients have a higher immunological risk than those undergoing their first transplantation [27] and were more common in the HLAi group. The amount of plasmapheresis was similar between HLAi and ABOi KT. HLAi KTRs used higher doses of rituximab than ABOi KTRs. It has been shown that anti-A/B antibodies has a lower effect on immunologic reaction in KT [9, 28, 29]. For the same reason, ATG as an induction therapy was used in HLAi and HLAi+ABOi KTRs, but not in ABOi KTRs.

The BPAR rate is a major concern in incompatible KTs. Our results showed excellent rejection-free survival and low occurrence rates of BPAR in both the HLAi and ABOi groups. A previous Korean multicenter study reported an incidence of BPAR of about 20% during 36 months of follow-up in HLAi patients [8]. Our study had a similar mean follow-up duration, but the incidence of BPAR was much lower in our study; only about 6% in the HLAi patients. This gap suggests that transplant centers have considerably different graft rejection rates and have different outcomes for incompatible KTs, even within the same country. HLA-DSA is known to be a stronger inducer for ABMR, especially early ABMR, than anti-A/B antibodies [29, 30]. We also produced similar results, finding that among BPARs the proportions of ABMR and early ABMR were higher in the HLAi group than in other groups. Although, there were no statistical differences in ABMR incidence due to the small number of occurrence, the incidence risk was higher in HLAi group and this could result in poor graft outcome in the long run. Long-term studies are needed in a large number of patients to clarify the risk of ABMR and graft survival in HLAi KT. However, Montgomery et al. [31] previously demonstrated HLAi KTRs had better survival than continuing dialysis patients. In addition, our study also showed that AMBR incidence was higher in the HLAi group but there was no significant difference compared to the control and LD control group. Taken together, HLAi KT would be a good treatment option for ESRD patients. In contrast to ABMR, the TCMR ratio was lower in the HLAi group, possibly related to the frequent use of induction ATG therapy. Several studies have shown that ABOi KT is at no higher risk for acute rejection than is non-sensitized KT [21, 32, 33], and the results of our study were consistent with this observation. We identified a similar incidence of BPAR between ABOi and control/LD control KTRs. It has also been shown that ABOi has no additive risk for acute rejection in HLAi KT [9], and no BPAR episode has occurred in our HLAi+ABOi KTRs.

Our patients showed a very low incidence of graft rejection not only in the ABOi and control groups, but also in the HLAi and HLAi+ABOi groups. The ABMR incidence in the HLAi group tended to be higher than in the control/LD control group, nevertheless, all KT groups had excellent graft function and graft survival. There was no difference observed in graft function and graft survival between any of the types of incompatible KTs and compatible KT. The observation period in our study was much longer than those in previous studies [4, 8], so we could demonstrate a good long-term prognosis for allografts in incompatible KTs. Using a multivariate Cox regression model, neither HLAi nor ABOi were risk factors for graft failure. Only donor age and any kind of rejection episode were identified as independent risk factors for graft failure in KTRs.

There were few deaths during the study period. Previous studies have demonstrated that neither HLAi nor ABOi are risk factors for infection, but it is associated with strength of desensitization [8, 34, 35]. It has also been suggested that tailored desensitization may reduce post-transplant infection and infection-related deaths [34, 35]. Our HLAi KTRs experienced more infections, such as urinary tract infections and CMV infections, than non-sensitized KTRs. High intensity desensitization, involving higher doses of rituximab and ATG induction, was used for HLAi KTRs, and these factors may have caused frequent infections. However, there was only one gastrointestinal infection-related death, at 66 months after KT, in the HLAi group. ABOi KTRs underwent relatively low-intensity desensitization, and did not show a significant increase in infection rates compared to the control group. There was also one gastrointestinal infection-related death, at 26 months after KT, in the ABOi group. These results imply that our desensitization protocol is not dangerous, and does not cause life-threatening infectious complications.

Previous studies evaluated the prognosis of HLAi and ABOi KTs [7–9]. However, there were no comparisons made with non-sensitized KT [7], there were small numbers of HLAi KTRs with short follow-up durations [9], and it was difficult to identify the exact immunological information and the outcomes produced by each transplant center [8]. We overcame these limitations, and our study demonstrates excellent outcomes for incompatible KTs. These results may help KT candidates and clinicians make decisions with regard to incompatible KTs.

There are some limitations in this study. The sample sizes of the HLAi, ABOi, and HLAi+ABOi KTs were relatively small, and there were few rejection, graft failure, and patient death events to identify statistical differences. The observation period was also relatively short, making it difficult to obtain definite results. In addition, except the LD control group, we analyzed the living donor and deceased donor KT prognoses together for each group. However, as shown in the Cox regression model, donor type did not appear to be a risk factor for graft failure.

In conclusion, the results from our center indicated that the transplant outcomes in all kinds of incompatible KTs—HLAi, ABOi, and HLAi+ABOi—were comparable to those of non-sensitized KTRs. Both incompatible KTs and compatible KTs had excellent outcomes in our hospital. Desensitization therapy did not cause life-threatening infectious complications. Our results indicate that incompatible KTs can be a safe treatment option for patients with ESRD.

## Supporting information

S1 Appendix Data set of the study. (XLSX)
